# Applying the results based management framework to the CERCA multi-component project in adolescent sexual and reproductive health: a retrospective analysis

**DOI:** 10.1186/s12978-018-0461-3

**Published:** 2018-02-08

**Authors:** Kathya Cordova-Pozo, Andrea J. Hoopes, Freddy Cordova, Bernardo Vega, Zoyla Segura, Arnold Hagens

**Affiliations:** 1 South Group, C. Ecuador O-138, Edificio Holanda, A-3A, Cochabamba, Bolivia; 2 Kaiser Permanente Washington, 13451 SE 36th, Bellevue, WA 98006 USA; 3 University of Cuenca- Facultad de Ciencias Médicas, Avenida 12 de abril S/N sector El Paraíso, Cuenca, Ecuador; 4 Instituto Centroamericano de la Salud, Reparto Los Robles, Restaurante La Marsellaise 1 c. al norte 1 c. al este, casa #, 77 Managua, Nicaragua

**Keywords:** Results based management, Multi-country, Community based interventions, Adolescents, Latin America, Sexual and reproductive health, Theory of change, Monitoring, Evaluation, Implementation research

## Abstract

**Background:**

Adolescent sexual and reproductive health (SRH), problems such as unplanned pregnancies are complex and multifactorial, thus requiring multifaceted prevention interventions. Evaluating the impact of such interventions is important to ensure efficiency, effectiveness and accountability for project funders and community members. In this study, we propose Results Based Management (RBM) as a framework for project management, using the Community Embedded Reproductive Health Care for Adolescents (CERCA) as a case study for RBM. The CERCA Project (2010-2014) tested interventions to reduce adolescent pregnancy in three Latin American countries, Bolivia, Ecuador and Nicaragua. Activities were designed to increase adolescent SRH behaviors in four domains: communication with parents, partners and peers; access to SRH information; access to SRH services; and use of contraception. When the project ended, the outcome evaluation showed limited impact with concerns about accuracy of monitoring and attrition of participants.

**Methods:**

We reviewed and analyzed a series of CERCA documents and related data sources. Key findings from these documents were organized within an RBM framework (planning, monitoring, and impact evaluation) to understand how CERCA methodology and performance might have reaped improved results.

**Results:**

Strengths and weaknesses were identified in all three elements of the RBM framework. In Planning, the proposed Theory of Change (ToC) differed from that which was carried out in the intervention package. Each country implemented a different intervention package without articulated assumptions on how the activities of intervention would bring about change. In Monitoring, the project oversight was mainly based on administrative and financial requirements rather than monitoring fidelity and quality of intervention activities. In Impact Evaluation, the original CERCA evaluation assessed intervention effects among adolescents, without identifying success and failure factors related to the outcomes, the nature of the outcomes, or cost-effectiveness of interventions.

**Conclusions:**

This analysis showed that multi-country projects are complex, entail risks in execution and require robust project management. RBM can be a useful tool to ensure a systematic approach at different phases within a multi-country setting.

## Resumen

**Antecedentes:**

La salud sexual y reproductiva de los adolescentes (SSR), como problemas de embarazos no planificados son complejos y multifactoriales, por lo que requieren múltiples intervenciones para su prevención. Evaluar el impacto de estas intervenciones es importante para asegurar la eficiencia, la eficacia y la rendición de cuentas para los donantes de proyectos y los miembros de la comunidad. En este estudio, proponemos la Gestión Basada en Resultados (o su sigla en inglés RBM) como un marco para la gestión de proyectos, utilizando el Community Embedded Reproductive Health Care for Adolescents (CERCA) como un caso de estudio para RBM. El Proyecto CERCA (2010-2014) puso a prueba las intervenciones para reducir el embarazo adolescente en tres países de América Latina, Bolivia, Ecuador y Nicaragua. Las actividades fueron diseñadas para mejorar los comportamientos adolescentes en SRH en cuatro aspectos: comunicación con padres, compañeros y pareja; acceso a la información sobre SSR; acceso a los servicios de SSR; y uso de anticonceptivos. Cuando el proyecto terminó, la evaluación de resultados mostró un impacto limitado sobre todo por la precisión en el monitoreo y pérdidas en el seguimiento de cohortes.

**Métodos:**

Se revisó y analizó una serie de documentos CERCA y fuentes de datos relacionados. Las principales conclusiones de estos documentos se organizaron en el marco lógico de la Gestión Basada en Resultados (planificación, monitoreo y evaluación del impacto) para comprender cómo la metodología y el rendimiento de CERCA podrían haber obtenido mejores resultados.

**Resultados:**

Se identificaron fortalezas y debilidades en los tres elementos del marco de RBM. En la Planificación, la propuesta Teoría del Cambio (ToC) difiere de la que se llevó a cabo en el paquete de intervención. Cada país implementó un paquete de intervención diferente sin supuestos articulados sobre cómo estas actividades de intervención producirían cambios. En el Monitoreo, la gestión del proyecto se basó principalmente en requisitos administrativos y financieros en lugar de supervisar la fidelidad y la calidad de las actividades de intervención. En la Evaluación de Impacto, la evaluación original del CERCA evaluó los efectos de la intervención entre los adolescentes, sin identificar los factores de éxito y fracaso relacionados con los resultados, la naturaleza de los resultados o el análisis de costo-efectividad de las intervenciones.

**Conclusiones:**

Este análisis mostró que los proyectos multinacionales son complejos, implican riesgos en la ejecución y requieren una gestión del proyecto sólida. La Gestión Basada en Resultados puede ser una herramienta útil para garantizar un enfoque sistemático en las distintas fases de un proyecto bajo un contexto multi-país.

## Plain English summary

Adolescent pregnancy can negatively affect the health and well-being of adolescents. Complex problems like adolescent pregnancy require multi-faceted prevention interventions that are carefully planned, implemented and evaluated in order to satisfy funders and community members. The Community-Embedded Reproductive Health Care for Adolescents (CERCA) Project (2010-2014) set out to lower adolescent pregnancy in Bolivia, Ecuador, and Nicaragua through multiple ways: increasing communication; increasing the number of adolescents who can get sexual and reproductive information and services, and increasing the number of adolescents using birth control. When the project ended, CERCA had not met all of its goals. In this paper, we studied the CERCA project using a specific project management framework called Results Based Management (RBM) to learn more about the project’s strengths and weaknesses. RBM is a system that can enhance effectiveness and verify that the project is going in the right direction using feedback loops at each level. Using the RBM framework, we found important lessons in the 3 main elements of RBM, which are (1) planning, (2) monitoring and (3) evaluation.

(1) We learned that what was proposed in the project differed from what was carried out. (2) We also learned that the way the project’s progress was monitored did not meaningfully allow for an understanding of what was being done. (3) We also learned that measuring the impact left some unanswered questions. This analysis using RBM sheds new light on lessons learned from the CERCA project and may be useful in future adolescent SRH initiatives.

## Background

Multi-component, community-integrated health intervention packages are necessary to address complex social problems such as adolescent pregnancy [9]. However, these intervention packages are often themselves complex and require intensive planning, sound implementation, and rigorous evaluation to ensure effectiveness, community acceptance, and return on investment by non-governmental organizations (NGO) and government agencies [16, 27]. Multi-country programmes have the opportunity to create broad, population-based change, but without the use of evidence-based approaches to create intervention packages and appropriate management of interventions, their impact may fall short ([11, 18, 22, 25].

In Latin America, the need for health intervention packages is particularly pressing in the area of adolescent sexual and reproductive health (SRH) as adolescent birth rates are among highest in the world [31]. The adolescent birth rate has remained persistently elevated, declining only from 77 to 72 per 1000 women of 15-19 years of age between 1990 and 2005, while access to contraceptive methods and comprehensive sexuality education remains restricted among adolescents [31]. Bolivia, Ecuador and Nicaragua face particularly high adolescent birth rates and associated poor health and psychosocial outcomes. In Bolivia, the adolescent birth rate is 89.1 per 1000 women (2005), Ecuador 99.6 (2002) and Nicaragua 92 (2010), thus are settings warranting special consideration for complex interventions [31].

A recent initiative titled Community-Embedded Reproductive health Care for Adolescents (CERCA) Project (2010-2014) aimed to reduce adolescent pregnancy rates and improve SRH of adolescents through a comprehensive strategy to modify health behaviors related to communication, SRH information-seeking, SRH care-seeking, and promotion of safe sexual relationships. To achieve these changes, CERCA implemented a strategy involving multiple stakeholders such as adolescents, parents, teachers, health personnel, health and education authorities, community and local authorities in three settings: Cochabamba (Bolivia), Cuenca (Ecuador) and Managua (Nicaragua).

CERCA was implemented from March 2010 and concluded in March 2014. The key institutions leading the implementation were The International Center of Reproductive Health-Ghent University in Belgium as the general project coordinator) and South Group in Bolivia, University of Cuenca in Ecuador, CIES and ICAS in Nicaragua, Kaunas University in Lithuania, and the University of Amsterdam in The Netherlands as the country implementers [4, 8]. The quantitative evaluation of CERCA, using a pre- and post-test controlled design, did not demonstrate effects after twenty months of intervention in the following key areas: reinforced behaviour, knowledge, attitudes, skills, and self-reflection. This lack of measured success was primarily attributed to challenges in data collection described in the CERCA report [16] amid others. A qualitative post hoc impact evaluation, supported by the World Health Organization, was carried out from June 2014 until March 2015 and aimed to expand upon the quantitative evaluation of the CERCA project (Crescendo [5]). This qualitative analysis revealed that a concerted application of a multi-faceted strategy reduced taboo in a conservative setting, sensitized the community and authorities, enabled the environment for adolescent interventions, and that subsequent, multi-country projects warrant a more thorough preparatory phase [13].

In the past, many health projects have been implemented without a clear idea of whether they did, in fact, achieve the expected results due to incomplete monitoring or inadequate evaluation phase [32]. However, more recently, a number of bilateral agencies and United Nations’ programs have adopted the Results Based Management (RBM) framework to demonstrate aid effectiveness and accountability from project planning to completion [1, 26]. We aimed to understand whether an RBM framework might have helped CERCA produce evidence of tangible and positive results by allowing country implementers to more effectively address challenges in design, implementation, monitoring, and evaluation.

The objectives of this paper are to apply the RBM framework retrospectively to the CERCA project in order to reflect on lessons learned and provide recommendations for future similar multi-country projects with complex, community-integrated interventions. This will be conducted through the application and analysis of three main components of RBM framework: 1) Planning, 2) Monitoring and 3) Impact evaluation. Findings may offer project managers, implementers, evaluators, and funding agencies valuable information regarding adolescent SRH intervention packages and improve effectiveness in management, accountability, and creation of evidence in subsequent SRH projects.

## Methods

RBM is a system designed to support efficiency and effectiveness at different stages of the project cycle starting from planning, designing the intervention package, monitoring, and supporting impact evaluation. RBM uses feedback loops at each of these levels. In complex interventions, this framework helps ensure that the project implements a package of interventions that is necessary and sufficient to achieve the expected result [29, 30]. Its goal is to enhance effectiveness and provide avenues to verify if the project is progressing appropriately through three phases: planning, monitoring and evaluation. *Planning* relies on a Theory of Change (ToC) framework which is the basis for the intervention package (IP). The ToC explains how a project will achieve a series of outcomes, by outlining causal relations based on assumptions to reach behavior change [15]. It involves choosing strategic interventions that are *necessary* and *sufficient* to reach the expected outcome [29, 30]. *Monitoring* tracks the process of change and how to ascertain success, tracking the implementation progress against the expected results of ToC [21]. Quantitative and qualitative evidence is collected for defined indicators to help determine the expected results from the intervention. *Impact evaluation* compares the control and intervention groups to assess the degree to which the impact can be attributable to the project after controlling for all external variables. Impact evaluation allows for decisions about whether project activities should be continued, replicated, scaled-up and improves the overall understanding about why certain changes have happened ([12, 21]. RBM is versatile enough to accommodate other research strategies within its qualitative arm such as *community-based participatory research* (CBPR). CBPR strategies foster empowerment in the community with the creation of knowledge as well as engagement, capacity building and quality assurance [10].

RBM can be used both retrospectively and prospectively to sharpen the strategic focus of the project. Organizations that use it for their project management are the Swiss Agency for Development and Cooperation and the United Nations Population Fund [24, 28]. In order to perform a retrospective analysis, we reviewed and analyzed a series of CERCA documents and other data sources (detailed in Appendix 1) structured by applying the RBM framework in order to see in what way the CERCA project performance might have reaped improved results. This included CERCA methodology design, final CERCA report 2014 (Crescendo [5]), internet report [23], post-hoc evaluation process report 2015 [16], international published papers [3, 4, 6–8, 13, 14, 17] and the A-form for results. Records and discussion notes from two meetings (recorded without transcript) were also reviewed: 3-day meeting in Cuenca-Ecuador (February, 2014), and 3-day meeting in Ghent-Belgium with country project leaders, researchers from Ghent and the study advisor from WHO-HRP (December, 2014).

## Results

### Planning

Findings related to planning and a theory of change (ToC) framework were divided into three key elements: 1) outputs, intermediate- and long-term outcomes, 2) identification of the preconditions to create change and of articulated assumptions, and 3) the intervention package, which contains a sequential pathway of change with indicators to assess the performance.

**Fig. 1:**
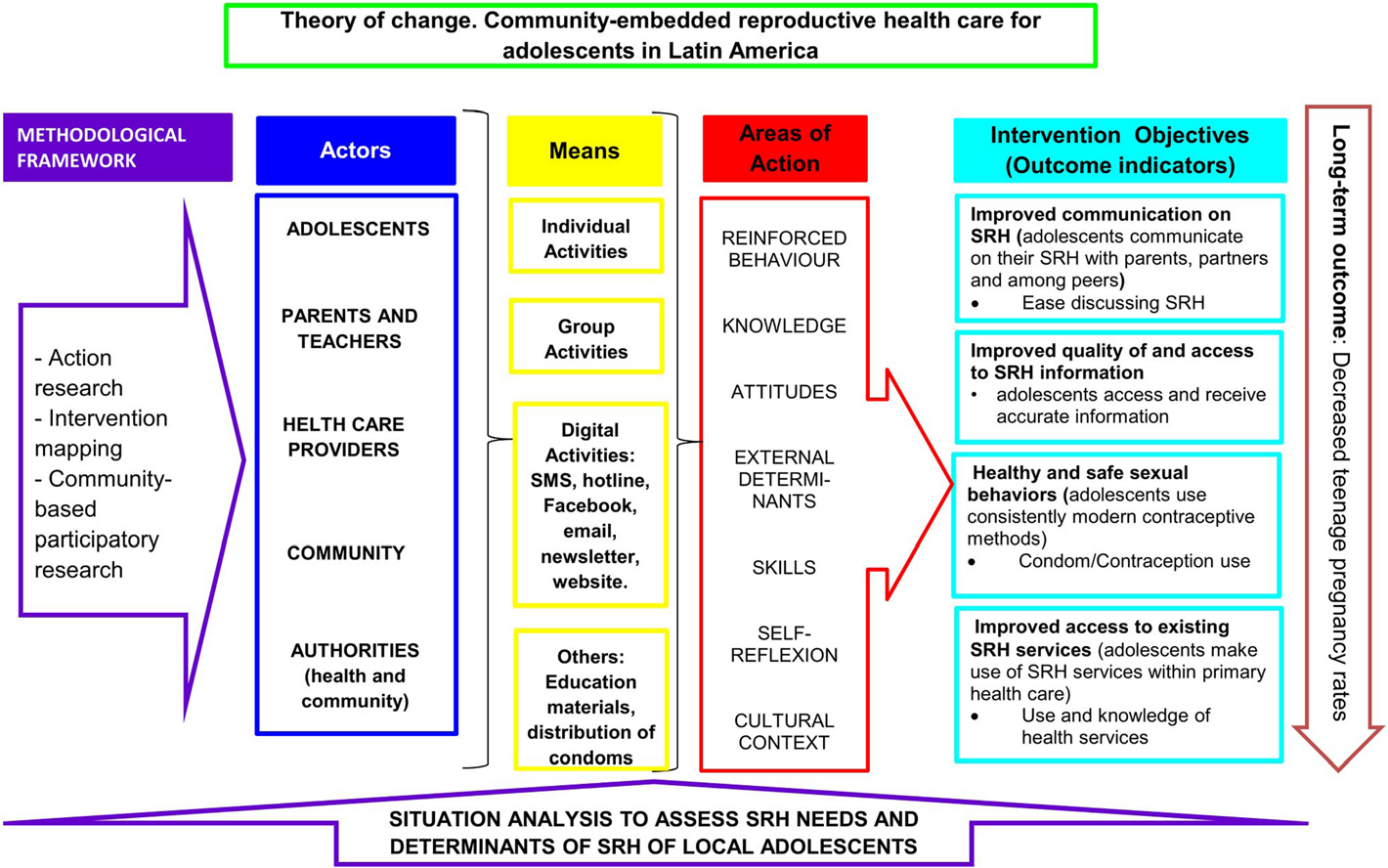
CERCA: Shortened version of the Theory of Change

**Fig. 2:**
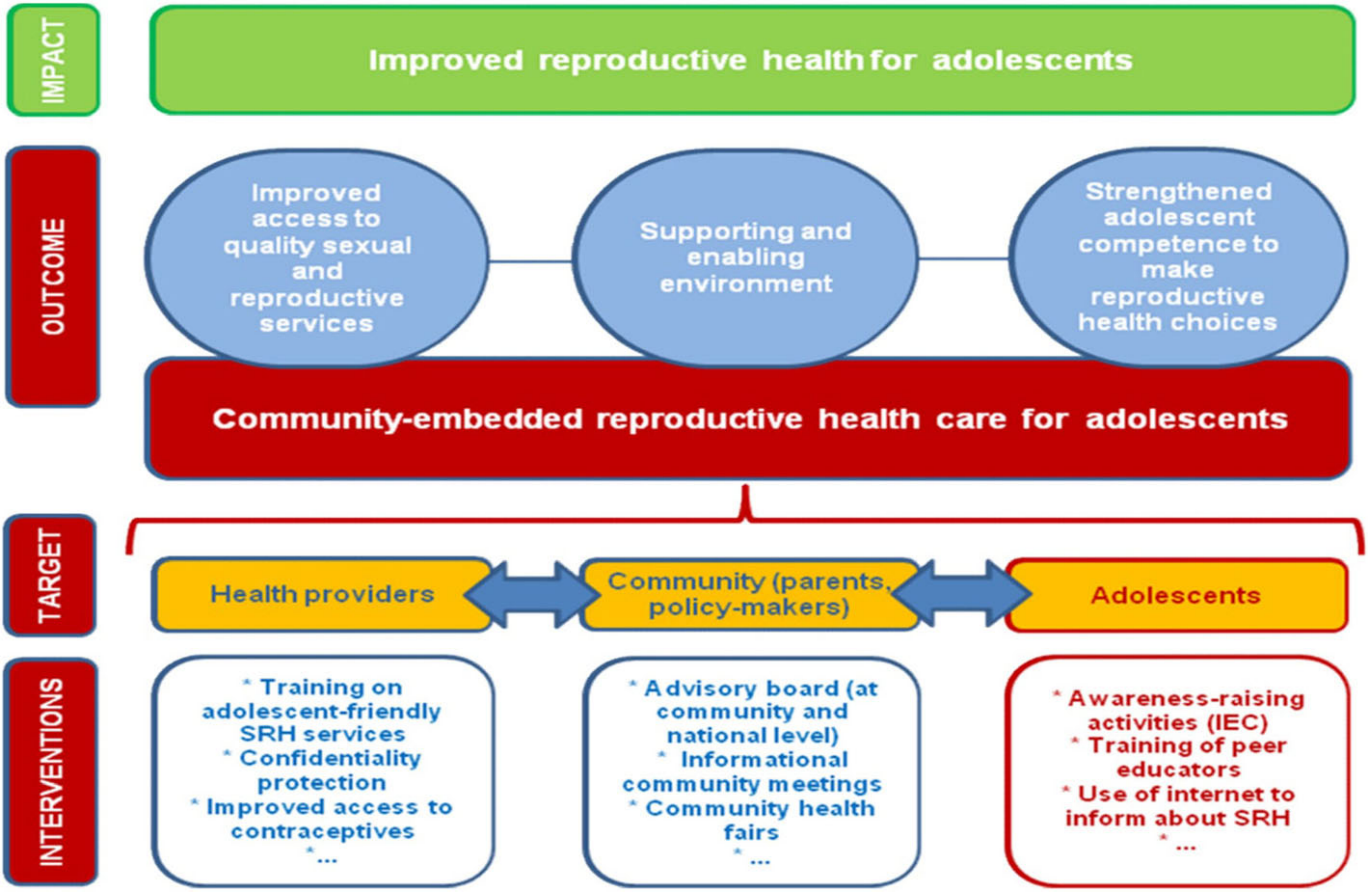
Plan: Expected impact of the CERCA project

1. *Outputs, intermediate- and long-term outcomes:* The CERCA project proposal included interventions targeting multiple outcomes, including improved access to quality sexual and reproductive services, a more supportive and enabling environment, and strengthened adolescent competence to make reproductive health choices (Fig. 1). The post-hoc evaluation revealed outcomes during the process of implementation, and a new ToC was developed based on the CERCA documents (Fig. 2) [13], more accurately reflecting what actually occurred in project implementation. This retrospective ToC was: a) flexible and could adapt to the needs of every country but included different outcomes than the ones originally defined in the proposal; b) did not display a causal analysis on how the activities of intervention would bring about change in a logical and chronological manner.
2. *Identification of the preconditions to create change and of the articulated assumptions:* Our review revealed that identification of preconditions and articulated assumptions did not occur due to construction of the ToC with different actors of the community. These community actors were involved from the beginning of the project until the end in order to promote credibility and buy-in with every activity. The ToC changed as activities were added and modified over time based on Community Based participatory research (CBPR) insights. This CBPR approach involved countries having exclusive meetings with every group of stakeholders without a common stakeholder meeting. This lead to the modification of country-specific interventions without adherence to the original ToC.

Although CERCA carried out two extensive data collections at the beginning of the project, the results were not used to identify articulated assumptions or preconditions to create change. One was a cross-sectional study of around 9000 adolescents aged 14-18 years old. The second was a qualitative study among health care providers. Due to the project size, there was insufficient time reserved for its analysis in depth; thus, this was deferred to the end of the project, by 2013 [14] and at the end of 2014 [7] consecutively.

3. *Intervention Package (IP)*. The IP was constructed by stakeholders using a CBPR approach but without a realistic timeframe to achieve the outputs and outcomes with each activity. Further, there is evidence that effective interventions were chosen based on literature review. There was no comprehensive guideline on how to design, implement or monitor the intervention activities (being the only ones found: SMS multi-country guideline and Guideline for SRH workshops in Bolivia). Each country had the flexibility to develop its own interventions and to determine the frequency of each activity based on the needs of target groups, CBPR insights, experience and disciplinary strengths of the local teams with different backgrounds [13].The IP was different in all three countries and not all activities were linked to the indicators of the intervention; hence, conditions to produce behavioral change were not created [30].

Table 1 and Appendix 2 demonstrates the variability of intervention activities in more detail. Table 1 demonstrates that: 1) each country had a different frequency of activities in every site; 2) interventions did not have a common pattern of activities in terms of coverage; 3) there are some IP features that could not be elicited with the reporting documents such as the duration of each activity or the how it was delivered. Appendix 2 demonstrates the finding that most activities focused in improving access to SRH information with less attention to the other three objectives of intervention: improved comfort discussing SRH; increased use of health services; and improved contraceptive use. Further, it shows that in Bolivia and Ecuador, the greatest emphasis was placed on workshops (in Bolivia 67% and Ecuador 81% of the total activity resources in the country were dedicated to workshops). Workshops contained the 4 objectives of intervention while in Nicaragua, 2 activities covered all the 4 objectives with 64% of the total activity resources. The percentage of coverage was under 100% in most of the activities, but in one case, coverage extended beyond to the target population by 639%.

**Table 1:** Bolivia - Ecuador - Nicaragua: Frequency of activities in each intervention site *

	School 1 Comm. 1	School 2 Comm. 2	School 3 Comm. 3	School 4 Comm. 4	School 5 Comm. 5	School 6 Comm. 6	Coverage and Observations while preparing the table
BOLIVIA: Frequency of activities for adolescents. Target 2000 adolescents in 12 schools							
Email on SRH	8	8	8	8	8	8	Reached: 500 that had email in the 12 schools
Bidirectional text messages	8	8	8	8	8	8	Reached: 1500 that had a phone in the 12 schools. More than 500 questions received
Workshops in SRH	5	5	5	5	5	5	Reached: 2000. 5 topics given to all the last 4 years of school. 30 adolescents per time × 5 times. In total 475 workshops done.
Private health consultations (Pilot study)	9	9	9	9	9	–	Reached: 350. Pilot for 6 months and was not implemented in the other treatment schools
ECUADOR: Frequency of activities for adolescents. Target 2000 adolescents in 3 schools							
Sport activities	2	2	3	na	na	na	Reached from school to school: 50, 40 and 1310 adolescents
Cinema on SRH	1	5	–	na	na	na	Reached from school to school: 40, 300 and 0 adolescents
SRH Fair	2	1	2	na	na	na	Reached from school to school: 2600, 800 and 1600 adolescents
Email on SRH	5	5	5	na	na	na	Reached from school to school: 841, 400 and 1000 adolescents
Text messages	1	1	1	na	na	na	Reached from school to school: 841, 400 and 1001 adolescents
Private health consultations	1	1	–	na	na	na	Reached from school to school: 18, 31 and 0 adolescents
Workshops in SRH	93	25	47	na	na	na	Frequency is not related to the # of topics. Reached from school to school: 10,048, 20,311 and 7988 adolescents
NICARAGUA: Frequency of activities for adolescents. Target 1500 adolescents in 6 communities							
FOY training to the community	51	36	46	69	58	52	Reached from comm. 2 comm.: 1020, 720, 920, 1038, 1160 and 1040 people
Forum cinema on SRH	21	23	23	19	23	29	Reached from comm. 2 comm.: 315, 345, 345, 285, 345 and 435 people
FOY training to couples	11	22	16	31	26	11	Reached from comm. 2 comm.: 22, 44, 32, 62, 52 and 22 parents, adolescents
Workshops done by ICAS	23	22	21	25	23	29	Frequency is not related to the # of topics. Reached from comm. 2 comm.: 345, 330, 315, 375, 345 and 435 teens.

* In Managua, the intervention work was done in communities, not in schools. Managua applied a cluster randomized controlled study in 6 communities. Interventions were carried out on schools in Cuenca (3) and in Cochabamba (12). This table displays only 6 of the schools in Cochabamba to make easier to compare

Source: Elaborated from documents of CERCA-A2 form

### Monitoring

Findings related to monitoring were organized into three key elements [1] [2]: 1) planning, 2) evaluation on how and to what extent the objectives are reached, 3) identification of failures to produce outputs.

1. *Planning*. We found no plan within the original CERCA documents to validate a monitoring approach. Furthermore, there was no pilot period to test whether the project was feasible or to check if the collected data was adequate to track change. Skill evaluations of the implementers following the intervention design were not found.

We learned that, due to a conservative society with taboos and machismo [4], country partners decided to first focus on activities to break down taboos and cultural gender issues, followed by activities to transmit awareness and sensitization about SRH. Although these steps were deemed necessary, they did not correspond with the ToC plan; thus, these activities were not monitored but still affected the timeframe of other planned activities (Ghent meeting).

2. *Evaluation of objectives*. The monitoring system envisioned by CERCA was mainly based on administrative and financial requirements (control per activity and trimester), rather than monitoring to track the level of fidelity to the intervention model, quality of service delivery or degree of behavioral change and community perception on SRH.

In addition, the coordination office prepared a multi-country monitoring template that gave priority to certain objectives of the ToC over others. For example, when reporting activities to authorities, the template tracked number of workshops, TV/radio presentations, meetings and people reached but not degree of sensitization or improvement of communication skills on SRH. Data collected for monitoring was not used for technical feedback (e.g. objective-outcome-correct path to reach change) that may have led to corrective actions. For instance, data was collected over 3 years at health care facilities to track the number of adolescents that visited the health care facility before, during and after the project but this data was never used to analyze if access to SRH services was improving. A project member in Ecuador suggested that both limited skills of some team members and inadequate monitoring templates contributed to evaluation challenges.

“…Not all members of the project knew how to monitor activities. There was a format to check options and that is what they did but did not register what was really done, did not tell the whole process of the activity…”

3. *Identification of failures.* In the absence of a monitoring plan, failures were not systematically identified; therefore, they were not reported. The 3-day meeting in Ghent revealed that when unexpected events occurred during interventions and corrective measures were taken, there was no report to account for changes that took place (Table 1 and Appendix 2). For instance, our analysis revealed that peer-to-peer activities (FOY) in Nicaragua reported the number of visits to adolescents per trimester, but did not report how many times each FOY visited each adolescent nor was there data on quality of interaction with each adolescent to evaluate its content and effectiveness for behavior change. We also found that open-air activities reported the number of participants but it did not detail if the persons stayed throughout the whole activity (to measure exposure to the intervention or implementation challenges) or if people returned to the next activity as monitoring only counted the participants. *A project member in Nicaragua proposed that there was resistance to monitoring forms* (Table 2)*.*
  “…Health promoters saw monitoring forms as something bad, as a way to control them, so they were filled as an obligation…”

**Table 2:** Failures of Monitoring

1) Planning
- No monitoring plan or no guideline to evaluate phase by phase
- No pilot to assess feasibility of intervention package, implementers’ skills, adequacy of data
- Interventions did not match predefined indicators
2) Evaluation on how and to what extent the objectives are reached
- Emphasis on administrative and financial monitoring
- Data collected focused only on number of activities and reached people; rather than quality of service delivery or degree of behavior change
- Single monitoring format for countries with different interventions
- Monitoring did not include all objectives of the ToC
- Data collected had no feedback on intervention activities
3) Identification of failures to produce outputs
- No monitoring to identify difficulties or flaws
- No monitoring to describe implementation challenges
- No report on mid-project adjustment of IP activities

### Impact evaluation

Our findings related to Impact Evaluation (IE) were also organized into three key elements, according to the RBM framework [21, 26]: 1) identification of success and failure factors to produce outcomes, 2) outcomes being positive, negative, anticipated or unanticipated, 3) cost-effectiveness, specifically the evaluation of the administrative and financial resources necessary to carry out the interventions.

1. *Identification of success and failure factors to produce outcomes:* The CERCA Consortium held monthly meetings designed to report out successful activities and key challenges. These meetings led to the addition or removal of some activities without corresponding adjustments to the monitoring plan or consideration of project framework. These changes varied by country site, but a comprehensive record of why changes were made was not maintained (e.g. Table 1 and Appendix 2). Many difficulties related to the project were revealed from the qualitative evaluation after the project had ended. In the qualitative evaluation, it was noted that qualitative findings were not integrated into monthly meetings or the final program evaluation. Rather, the qualitative research was conducted separately, preventing responsive changes in the intervention based on its findings.
2. *Outcomes assessed as positive, negative, anticipated or unanticipated.* Not all outcomes were assessed within an adequate time frame to allow for change. For example, reducing the pregnancy rate in adolescents was established as a main outcome (Fig. 2). However, based on existing frameworks of pregnancy prevention interventions, 20 months may be too short of time frame to measure impact [19]. Some positive outcomes were not anticipated, such as a reduction of taboo in a conservative setting through concerted application of multi-faceted strategies, greater sensitization of community to an enabling environment [13]. This also promoted the expansion of SRH networks in Ecuador and Bolivia [13]. CERCA tested and evaluated SMS, phone, and social media to improve communication between health provider and adolescents and within networks of professionals and authorities by using social media, newsletters and websites [3, 23]. These were found to be effective strategies to foster open communication about SRH despite very conservative cultural norms [17]. Among negative outcomes were that parents did not participate in activities in as high numbers as expected or intervention emails had less than 10% opening rate by participants. Some outcomes were only evaluated at the end of the project using data from a self-administered survey. Survey results revealed challenges in the collection and interpretation of the data (attrition, type of questions, cohort coding); and in the implementation of some interventions [(low rates of follow up, high rotation of personnel (in the case of the friends of youth (FOY)] that may have led to unexpected results
3. *Cost-effectiveness: evaluation of the administrative and financial resources required to provide the interventions.* There was no plan to assess cost-effectiveness in every intervention or as one multi-component intervention. FOY was considered one of the most heavily promoted interventions of CERCA in Nicaragua but there is no cost-effectiveness evaluation to support this. Personnel of FOY worked for minimal reimbursement in the project, but there is no evaluation if this action would be feasible to scale-up at national level. Further Nicaragua experienced high turnover of personnel (6 months in average) suggesting that staff would likely not stay permanently in a volunteer position, which would hinder its scalability. The aspect that was evaluated for cost-effectiveness was the SMS intervention. The published evaluation reported cost per message, but did not include the cost of the personnel involved or the infrastructure with the software needed (internet and software, type of phone, hours of the personnel) [3]. This limited analysis creates difficulty assessing its feasibility for continuity or scalability at national level.

Based on the results of CERCA, a summary of the success and failure factors are summarized in Table 3.

**Table 3:** Impact evaluation: success and failure factors in CERCA

Success	Failures
1) Bolivia and Ecuador: Achieved modest behavioral change	1) Data collected at t0 and t1 only for adolescents and not other stakeholders
2) Reduced taboo, increased sensitization on SRH	2) Inaccuracies in survey data collection
3) Organized and improved community participation	3) Not all data collected was used in the impact evaluation
4) Bolivia and Ecuador: Expanded SRH networks	4) Necessary first steps were omitted in the ToC; thus, not evaluated
5) Tested Information and communication technology (ICT) to increase communication in SRH	5) No multi-country intervention package but a common monitoring format that prevented good monitoring and evaluation
6) Contributed to multi-country project design	6) Cost-effectiveness was not assessed. Not all the activities were evaluated due to time and budget restrictions.

## Discussion and lessons learned

The CERCA programme relied upon three methodological frameworks: action research, community based participatory research and intervention mapping but did not adopt any project management framework. When the project ended, CERCA had not met all of its goals. Using the RBM framework, we expand the CERCA insights to demonstrate the importance of appropriate timeframes and cause-effect analyses while planning activities that can originate both from the community and from review of effective, evidenced-based interventions. Testing the activities for feasibility and acceptability in a pilot can enhance a project’s chances of success. Designing a clear monitoring plan for each activity, reporting failures during the process, and having constant feedback meetings on the advancement process against the ToC is paramount. Evaluation should assess cost-effectiveness of every intervention, success and failure factors to produce outputs and outcomes, and a breakdown analysis to measure the progress at the end of the project so that future initiatives can benefit of this information. This analysis conveys in a summary of lessons learned (Table 4).

**Table 4:** Summary of lessons learned with the RBM analysis

Summary of lessons learned
*1) Planning: theory of change*
- Review efficient interventions, what-why-how they worked
- Beware of time restrictions and cause-effect analysis for every activity of intervention
- Conduct a pilot intervention to test the intervention package within the RBM framework
- Keep a common intervention package and add activities when needed case-by case
- Make a specific plan for community participation based on CBPR
*2) Monitoring*
- Design guidelines for every activity to ensure effective delivery of the content
- Monitor collection of data, phase of every activity, failures
- Standard monitoring is useful when same intervention package across multiple sites
- Obtain feedback on positive and negative aspects of IP and Monitoring
- Monitor all aspects (positive/negative) of each activity
*3) Impact Evaluation*
- Analyze success and failure factors to produce outputs/outcomes
- Evaluate the cost-effectiveness of every intervention
- Conduct a breakdown analysis of every intervention to understand the progress and particularities
- Preview time and budget to analyze lessons and determine the effective interventions

Multi-component, community-integrated health intervention packages are necessary to address complex social problems such as adolescent pregnancy [9]. This analysis applied an RBM framework retrospectively in order to learn from a multi-country project to improve adolescent sexual and reproductive health. The analysis using the RBM framework highlights important findings of the project. In *Planning*, the proposed Theory of Change (ToC) included a range of stakeholders who did not consistently plan activities based on evidence, situation analysis, timeframe or a sequential path of change. Intervention packages changed according to different circumstances and did not always take into account guidelines or quality of delivery, leading to different results across sites which were difficult to compare. Some planned activities did not take into account the possibility to be continued, reproduced or scaled-up. We identified missed opportunities to select evidence-based interventions with appropriate linkages to a ToC that would ensure the intended outcomes were reached. In *Monitoring,* we also highlighted gaps due to time, resources, and variability across sites. The project oversight was mainly based on administrative and financial requirements rather than monitoring fidelity or challenges to carry out the intervention model regarding content, behavior change, quality of delivery and community perception. In *Impact Evaluation*, the project did not plan a comprehensive evaluation, did not dedicate sufficient time and budget. Limited data was available due to inadequate record keeping; the original evaluation did not identify success and failure factors related to the outcomes, and the cost-effectiveness analysis was not inclusive of all activities and costs (e.g. analysis did not account for training time and costs, challenges to monitoring their activities and their impact on every adolescent from the intervention group). Examining the project within an RBM framework generates valuable insight for future adolescent health initiatives.

Research is growing in the area of project accountability and assessing validity of evidence from complex interventions Our analysis with RBM is an effective accountability mechanism that controls at all levels and can strengthen management of large-scale projects that include cross-sectoral strategies. The use of RBM from the start to the end of the project encourages managers to make decisions with the results in mind and to plan all aspects of the project in advance with detail, essential for complex interventions [29]. Over the years, many NGOs and other institutions implementing health projects in the field of SRH have focused more on their respective donors’ needs and not on how the project contributes to a broader social change [20]. With the implementation of RBM in international institutions, donors are encouraged to support implementation as well as data collection, evaluation and sharing of information gathered. In the case of CERCA, project leaders had the opportunity to evaluate various factors of success and failure under the supervision of an advisor from the WHO-HRP who oriented these talks and helped understand the lessons learned (meeting in Ghent, 2014); but not all projects have this opportunity as most do not even have budget and time planned for this.

The RBM framework analysis has some limitations. Firstly, this is a retrospective assessment and CERCA did not use it in any phase. Secondly, our analysis relied upon the availability and quality of records kept during planning, implementation and evaluation (Appendix 1). Third, the authors of this paper were the project managers or project implementers in every country; therefore, this may introduce bias into the analysis. Finally, this analysis was conducted retrospectively after the qualitative evaluation and may be influenced by these findings.

This analysis demonstrates the delicate balance between planning comparable multi-country interventions and allowing each country to develop its own approach per their specific needs. A thoughtful project design incorporates a situation analysis of the unique needs of each site together with scientific evidence on what interventions have been shown to work and how they might work in each context. More research is needed in this area to evaluate the feasibility and effectiveness of complex intervention packages.

## Conclusions

This analysis showed that multi-country projects with cross-sectoral strategies are complex, entail risks in execution and require rigorous project management. Large-scale, multi-country programmes have the opportunity to create broad, population-based change, but without the use of evidence-based approaches and appropriate management of interventions, their impact may fall short. RBM can be an effective accountability mechanism to ensure a systematic approach and appropriate use of resources at different levels within a multi-country setting. RBM can offer country implementers and managers an avenue to apply systematic management at all levels, improve accountability, promote the development of evidence base and strengthen commitment to adolescent health in future initiatives.

## Appendix 1

**Table 5:** Reviewed documents, classified by category and key elements of planning (1), monitoring (2), and impact evaluation (3)

*(1) DOCUMENTS USED FOR PLANING:*	*Reviewed for key element*	
CERCA Project proposal document, 2009	1	
Crescendo Stat-Ghent, Quantitative evaluation of the CERCA Project. Ghent University. 2014	1	
Ivanova O. et al. Lessons learnt from the CERCA Project, a multicomponent intervention to promote adolescent sexual and reproductive health in three Latin America countries: a qualitative post-hoc evaluation. Eval Program Plann-ELSEVIER. 2016, Jun13;58:98-105.	1, 2, 3	
Minutes of Cuenca meeting. 3-day meeting with country project leaders of the Consortium (February, 2014)	2, 3	
Minutes and recordings of Ghent meeting. 3-day meeting with country project leaders, researchers from Ghent and the study advisor from WHO-HRP (December, 2014)	2, 3	
Rojas Salazar M., Informe ESSA 2012, Análisis estadístico. En Revista Proyecto CERCA N°2. Estadísticas de Salud Sexual y Reproductiva en adolescentes Análisis de sus determinantes y Guía de Atención psicológica. Cochabamba	2	
Jaruseviciene L., Orozco M., Ibarra M., Cordova-Ossio F., Vega B., Auquilla N., Medina J., Gorter A., Decat P., De Meyer S., Temmerman M., Edmonds A., Valius L., Lazarus J. Primary healthcare providers’ views on improving sexual and reproductive healthcare for adolescents in Bolivia, Ecuador, and Nicaragua. Glob Health Action. 2013; 6: 10.3402/gha.v6i0.20444. Published online 2013 May 15. doi: 10.3402/gha.v6i0.20444. PMCID: PMC3656216.	2	
Decat, P. et al., 2015. Sexual onset and contraceptive use among adolescents from poor neighbourhoods in Managua, Nicaragua. Eur J Contracept Reprod Health Care., Mar 4; 20(2)(doi: 10.3109/13625187.2014.955846), p. 88–100.	2	
SMS-Multi-country guidelines, 2010	3	
SRH workshops guidelines for Bolivia, 2010	3	
CERCA-A2 form: List of activities done per country, 2014	3	
(2) DOCUMENTS USED IN MONITORING:	Reviewed for key element	
Cordova-Pozo K. et al., Improving Adolescent Sexual Health in Latin America: reflections from an International Congress. Reproductive Health.2015, 12:11. DOI: 10.1186/1742-4755-12-11	1	
Minutes and recordings of Ghent meeting. 3-day meeting with country project leaders, researchers from Ghent and the study advisor from WHO-HRP (December, 2014)	1, 3	
Monitoring: Report per activity	2	
Monitoring: Summary of Activities per trimester	2	
Monitoring: Summary of Activities per semester	2	
Data collected at health care facilities from 2010 to 2013, to track the number of adolescents that visited the health care facility	2	
Manuscripts for quality evaluation to CERCA from key informants: 2014	3	
(3) DOCUMENTS USED IN IMPACT EVALUATION:	Reviewed for key element	
Minutes of Cuenca meeting. 3-day meeting with country project leaders of the Consortium (February, 2014)	1	
Minutes and recordings of Ghent meeting. 3-day meeting with country project leaders, researchers from Ghent and the study advisor from WHO-HRP (December, 2014)	1	
CERCA-A2 form: List of activities done per country, 2014	1	
CERCA Project proposal document, 2009	2	
Ivanova O. et al. Lessons learnt from the CERCA Project, a multicomponent intervention to promote adolescent sexual and reproductive health in three Latin America countries: a qualitative post-hoc evaluation. Eval Program Plann-ELSEVIER. 2016, Jun13;58:98-105.	2	
Assessment report on internet for awareness raising activities, South Group, CERCA Project. 2014.	2	
Cordova-Pozo K. and Hagens A.J.J., Information and Communications Technology in Sexual and reproductive health care for Adolescents - A Bolivian Case Study. In Proceedings of the 2nd Annual Global Healthcare Conference: 2014; Singapore. Published by GSTF. July, 2013. ISSN: 2251-3833. Singapore	2, 3	
Nelson, E., Edmonds, A., Ballesteros, M., Encalada Soto, D., & Rodriguez, O. The unintended consequences of sex education: an ethnography of a development intervention in Latin America. Anthropology & Medicine, 21(2), 189-201. DOI: 10.1080/13648470.2014.918932	2	
Michielsen, K. et al., Effectiveness of complex interventions: Post hoc examination of the design, implementation and evaluation of the CERCA project to understand the results it achieved, Ghent: ICRH, Ghent University. 2015	2	
CERCA Project proposal document, 2009	3	

## Appendix 2

**Table 6:** Objectives of activities per country, percentage of effort and coverage

Intervention Package	Objectives of Intervention					
	Improve access and reception to accurate information	Improve Ease discussing SRH	Improve condom contraception use	Improve use of health services	% of effort*	% of coverage **
BOLIVIA: Activities for adolescents. Target 2000 adolescents in 12 schools						
Email on SRH	x				13.5%	25%
Bidirectional text messages (ICT)	x	x			13.5%	75%
Workshops in SRH	x	x	x	x	67%	100%
Private health consultations (Pilot study)	x	x	x	x	6%	18%
*% of effort per planned objective:*	*36%*	*27%*	*18%*	*18%*	*100%*	
ECUADOR: Activities for adolescents. Target 2000 adolescents in 3 schools						
Sport activities	x				3%	23%
Cinema on SRH	x				3%	6%
SRH Fair	x				3%	8%
Email on SRH (ICT)	x				7%	4%
Text messages (ICT)	x	x			2%	4%
Private health consultations	x	x	x	x	1%	0.8%
Workshops in SRH	x	x	x	x	81%	639%
*% of effort per planned objective:*	*50%*	*21%*	*14%*	*14%*	*100%*	
NICARAGUA: Activities for adolescents. Target 1500 adolescents in 6 communities						
FOY training to the community	x	x	x	x	44%	65%
Foro cinema on SRH	x				19%	23%
FOY training to couples	x	x	x	x	17%	3%
Workshops done by ICAS	x	x	x	x	20%	20%
*% of effort per planned objective:*	*31%*	*23%*	*23%*	*23%*	*100%*	

* % of effort was calculated by adding the total number of times each activity was carried out over the total activities in the country

** Coverage is calculated as the percentage of those in the target age group (adolescents) who were effectively reached with each activity

Source: Elaborated from documents of CERCA-A2 form

## Abbreviations

CBPR: Community-based participatory research
CERCA: Community-embedded reproductive health care for adolescents
ICT: Information and communications technology
IE: Impact evaluation
IP: Intervention Package
RBM: Results Based Management
SMS: Text messages
SRH: Sexual and reproductive health
STI/HIV/AIDS: Sexually transmitted infections / Human Immunodeficiency Virus / Acquired Immunodeficiency Syndrome
ToC: Theory of Change
WHO-HRP: The World Health Organization’s Department of Reproductive Health and Research
